# Natural History of Auditory Function in Patients with Alport Syndrome: A Case Series Study

**DOI:** 10.3390/jcm13226639

**Published:** 2024-11-05

**Authors:** Juyun Nam, Hyuntaek Jung, Dongju Won, Heon Yung Gee, Jae Young Choi, Jinsei Jung

**Affiliations:** 1 Department of Otorhinolaryngology, Yonsei University College of Medicine, Seoul 03722, Republic of Korea; juyunnam@yuhs.ac (J.N.); jht7072@gmail.com (H.J.);; 2 Department of Laboratory Medicine, Yonsei University College of Medicine, Seoul 03722, Republic of Korea; wdjbabo@yuhs.ac; 3 Department of Pharmacology, Graduate School of Medical Science, Brain Korea 21 Project, Yonsei University College of Medicine, Seoul 03722, Republic of Korea; hygee@yuhs.ac

**Keywords:** Alport syndrome, sensorineural hearing loss, pure tone audiometry, X-linked Alport syndrome, Autosomal Dominant Alport syndrome, Autosomal Recessive Alport syndrome

## Abstract

**Background**: Alport syndrome (AS) is a genetic disorder characterized by progressive renal disease, ocular abnormalities, and sensorineural hearing loss. However, the audiological profile of patients with AS remains elusive. Thus, this study aims to evaluate the natural history of auditory function in patients with AS. **Methods**: Exome or targeted sequencing for deafness genes was performed to confirm the pathogenic variants in patients with AS. **Results**: We identified fifteen individuals with AS who carried pathogenic variants of *COL4A3*, *COL4A4*, or *COL4A5*. Among fifteen, twelve (80%) showed hematuria, and six (40%) showed proteinuria. The patients exhibited bilateral sensorineural hearing loss, which was progressive and symmetric. The hearing thresholds increased according to age and plateaued at the level of 53 dB HL, indicating the hearing loss did not reach the severe-to-moderate level. The auditory dysfunction showed a distinct natural history depending on the inheritance pattern, but there was no remarkable difference between males and females among X-linked AS. **Conclusions:** Auditory dysfunction in AS is progressive up to the level of moderate hearing loss. Precise auditory rehabilitation for patients with AS is warranted depending on the inheritance pattern and genetic predisposition.

## 1. Introduction

Alport syndrome (AS) is a hereditary disorder primarily affecting the kidneys, eyes, and hearing, caused by mutations in the genes encoding type IV collagen [1,2,3]. The condition is characterized by progressive renal failure, ocular abnormalities, and sensorineural hearing loss (SNHL), which together form the hallmark triad of the syndrome [4,5,6]. Genetic mutations in the *COL4A3*, *COL4A4*, and *COL4A5* genes lead to the defective formation of the glomerular basement membrane (GBM) in the kidneys, as well as the cochlear basement membrane, resulting in the primary clinical manifestations of the disease [6,7,8]. Although the prevalence of AS is uncertain, screening urinalyses in the school-age population have identified a number of affected patients [1]. Given that the progressive pattern of AS is typical and that early treatment guarantees a delay in the progression to kidney failure [9,10,11], early identification and treatment of affected patients are prerequisite to maintain nephrological and audiological functions and improve the quality of life of patients with AS [1].

GBM is composed of collagen type IV, which consists of six different alpha chains (α1–α6) [12,13]. Among them, three subunits form triple helix structures. The combination of alpha chains is organ-specific. For instance, α3- α4- α5 is expressed in the GBM of the cochlea and ocular lens, whereas α5- α5- α6 is expressed in the Bowman’s capsule in the kidney [14]. If one of the alpha helix chains is absent or misfolded, the triple helix structures are disrupted, leading to pathogenic changes in the GBM, which subsequently cause nephropathy and SNHL. In particular, the stria vascularis, an essential part of the cochlear duct, requires intact type IV collagen to maintain ionic balance and fluid homeostasis in the inner ear [15,16]. Damage to the basement membranes in the cochlea can result in progressive hearing loss [17].

The inheritance pattern of AS is primarily X-linked, resulting from mutations in the *COL4A5* gene, which accounts for approximately 80% of cases [18]. Autosomal recessive (AR) and autosomal dominant (AD) forms of the disease are caused by mutations in the *COL4A3* and *COL4A4* genes, accounting for the remaining cases [19]. The X-linked form (XL) is generally more severe in males, who experience earlier onset of renal failure and more pronounced hearing loss due to the absence of a compensatory normal allele. Females with XL-AS typically exhibit a milder course due to random X-chromosome inactivation, although some may still develop significant renal and audiological complications [1,20].

Hearing loss in AS is one of the most recognizable phenotypic traits and often manifests in childhood or adolescence. SNHL in these patients tends to affect high frequencies and can progressively worsen, ultimately leading to significant impairment. Understanding the correlation between specific genetic mutations and the onset and severity of hearing loss is critical for improving the clinical management and prognosis of patients with AS [14]. Audiological assessments, such as pure-tone audiometry, are commonly used to monitor hearing thresholds and assess the degree of hearing loss in affected patients.

While the link between genetic mutations in the collagen IV genes and renal disease in AS is well-established, the genotype–phenotype correlation for hearing loss remains less understood. In general, severe, early-onset hearing loss is most commonly associated with XL-AS in males and AR-AS, while milder late-onset hearing loss is often seen in female carriers of XL and AD-AS, where the mutations allow for some residual collagen function [14]. However, the specific mutation type and the associated collagen gene further modulate the severity and timing of the auditory impairment. Recent studies have shown a significant variability in the degree and progression of renal diseases in AS depending on the affected causative genes, suggesting that other genetic or environmental factors may modulate the audiological phenotype [21]. This variability makes it imperative to study the relationship between specific mutations and audiological outcomes in greater detail.

This study aims to investigate the genotype and audiological phenotype correlation in a cohort of patients with AS using a dataset that includes both genetic and audiological information. We hypothesize that exploring the genotype–phenotype relationship in AS could provide information about the severity and progression of hearing loss. By analyzing the impact of various mutations in the *COL4A3*, *COL4A4*, and *COL4A5* genes, we seek to identify patterns that could help predict hearing loss severity and progression. Ultimately, understanding these genotype–phenotype correlations could improve the early detection and management of hearing loss in patients with AS.

## 2. Materials and Methods

### 2.1. Patient Enrollment

The current study included individuals exhibiting AS phenotypes, including proteinuria, hematuria, and hearing loss. The inclusion criteria were patients who were genetically confirmed to have pathogenic or likely pathogenic variants causing AS. The individuals were enrolled from the Yonsei University Hearing Loss (YUHL) [22,23,24] cohort. All patients in the YUHL cohort were audiologically screened to identify those with hearing loss. When a family history was noted, the available family members also underwent audiological testing. Genetic tests were performed for the affected patients and their family members when a genetic form of hearing loss was suspected. This protocol was followed for all the enrolled patients in this study. A total of 15 individuals participated in this study, comprising four men (26.7%) and twelve women (73.3%), with a mean age of 38.7 ± 19.9 years. The study received approval from the Institutional Review Board of our hospital (approval no. 4-2015-0659), and written informed consent was obtained from all participants.

### 2.2. Evaluation of Hearing Function

All the enrolled patients and their family members undertook pure-tone audiometry, as previously reported [25,26,27]. Briefly, the pure-tone air (500–4000 Hz) and bone conduction (500–4000 Hz) thresholds were measured using clinical audiometers in a double-walled audio booth. The hearing threshold was calculated as the average threshold at 500, 1000, 2000, and 4000 Hz (PTA_4_). The hearing loss was classified into mild (25 ≤ PTA_4_ < 40 dB HL), moderate (40 ≤ PTA_4_ < 70 dB HL), and severe-to-profound (PTA_4_ ≥ 70 dB HL) levels.

### 2.3. Genetic Analysis

Genetic testing using next-generation sequencing was performed for individuals and their family members, as previously reported [23,24,28]. A two-track approach to genetic testing was implemented, utilizing either whole-exome sequencing (WES) or a deafness gene panel, depending on the patient’s insurance coverage and their preference. For panel-based next-generation sequencing, a customized 207-gene deafness panel, as previously described [23], was employed. WES was performed using the Agilent SureSelect V5 enrichment capture kit (Agilent Technologies, Santa Clara, CA, USA), following the manufacturer’s protocol for sample preparation. Sequencing was conducted on the MiSeq platform (Illumina, San Diego, CA, USA) with the MiSeq Reagent Kit v2 (300 cycles) for high-throughput sequencing. Sanger sequencing was used for segregation analysis. Variants were identified using the “Basic Variant Caller” function in CLC, requiring a minimum of five reads, 20x coverage, and a frequency of at least 20%. Variants with minor allele frequencies exceeding 0.5% for recessive and 0.05% for dominant hearing loss genes in the dbSNP and gnomAD databases were excluded. Genetic diagnoses were confirmed by a multidisciplinary board of otolaryngologists and clinical geneticists, adhering to the hearing-loss-specific guidelines from the American College of Medical Genetics and Genomics (ACMG) and the Association for Molecular Pathology (AMP), as referenced in the Deafness Variation Database [29].

### 2.4. Statistical Analysis

All analyses were performed using Prism v8.0 (GraphPad Software, San Diego, CA, USA).

## 3. Results

### 3.1. Clinical Characteristics of Patients with AS Variants

We analyzed the clinical and genetic characteristics of 15 patients with pathogenic variants of the *COL4A3*, *COL4A4*, and *COL4A5* genes. The patient demographics are shown in Table 1. The age of onset varied, ranging from the first to the fifth decade. The hearing impairment was bilateral and sensorineural. The average thresholds for the pure-tone audiogram in the right and left ears were 31.3 ± 19.6 dB and 38.2 ± 20.2 dB, respectively, suggesting mild hearing loss in both ears. Although four patients (10.0%) had normal hearing at a pure-tone average, their age ranged from 3 to 16 years, which was too young to exhibit the auditory phenotype yet.

### 3.2. Genetic and Audiological Features in Patients Diagnosed with AS

Among the 15 affected patients, fourteen pathogenic variants were identified. The variants are classified into “pathogenic” or “likely pathogenic” in either DVD classification or ACMG/AMP guidelines. Of these, four variants were novel and are reported here for the first time (Table 2); the novel variants include c.1029+1G>A (COL4A3), c.3985C>T (COL4A5), c.584G>T (COL4A5), and c.3860_3861dupAA (COL4A5) and have been classified as pathogenic or likely pathogenic according to the DVD classification or ACMG/AMP guidelines.

**Figure 1:**
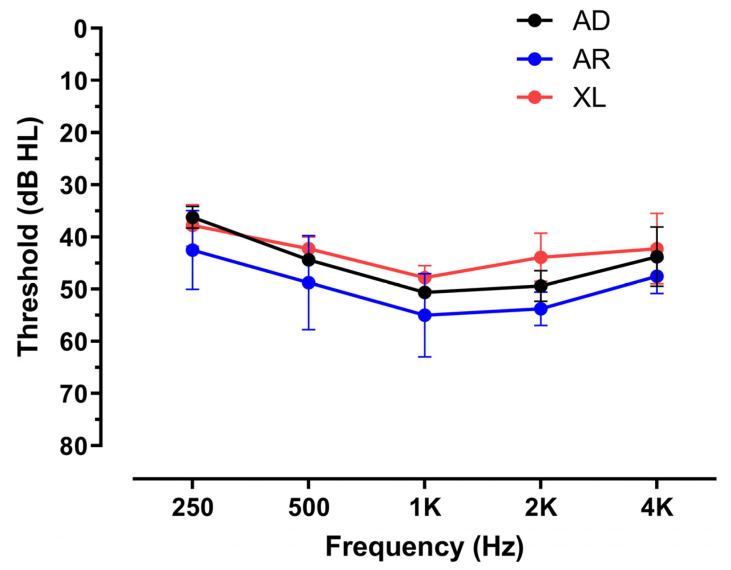
Audiograms of patients with pathogenic variants of the *COL4A3*, *COL4A4*, or *COL4A5* genes. The pure-tone audiograms of 15 individuals with pathogenic variants are depicted. The auditory thresholds were compared across groups using a two-way ANOVA test followed by multiple comparisons. There was no significant difference in the thresholds between the three groups at each frequency. The auditory thresholds indicate the average value of the PTA_4_ thresholds in both ears. AD, autosomal dominant type; AR, autosomal recessive type; XL, X-linked type.

Next, the patients were categorized into three groups according to genetic inheritance patterns. Then, the PTAs were compared to each other. In terms of the results, the audiogram patterns between the three groups were similar without a significant difference (Figure 1). The patterns were flat-type, and the thresholds ranged from 30 to 60 dB H, which indicated moderate level hearing loss.

### 3.3. Progression of Hearing Loss in Patients with AS

**Figure 2:**
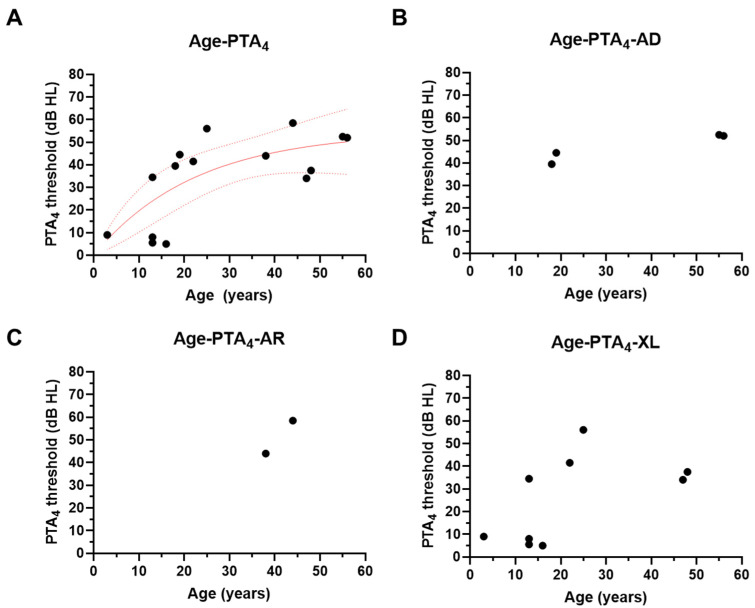
Progression of hearing loss in patients with AS. (**A**) The PTA4 thresholds are depicted as a function of age (*n* = 15). The red line refers to the nonlinear fitting model, and the dashed line refers to the 95% confidence line of the fitting model (exponential plateau fitting model, Y = Y_M_ − (Y_M_ − Y_0_) × exp(−k × x); Y_M_ = 54.72; Y_0_ = 0.00; k = 0.04, r^2^ = 0.49). (**B**–**D**) The PTA_4_ thresholds are drawn as a function of age in the AD-AS (**B**), AR-AS (**C**), and XL-AS (**D**) subgroups. The auditory thresholds indicate the average value of the PTA_4_ thresholds in both ears. AD, autosomal dominant type; AR, autosomal recessive type; XL, X-linked type.

The progression of hearing loss was evaluated through pure-tone audiometry (PTA) among the patients diagnosed with AS. In the 15 individuals, the PTA_4_ showed a gradual worsening of hearing thresholds as the age increased, with a significant rise in the PTA values starting around the age of 20. This trend indicates progressive SNHL, becoming more severe in older patients. However, the thresholds plateaued to the level of 53 dB HL according to the nonlinear fitting model and did not reach above 60 dB HL (Figure 2A). Nonlinear regression analysis of the hearing loss revealed that mild hearing loss began in the early teens, while moderate hearing loss started in the late 20s. Next, the PTA thresholds were analyzed across different genetic inheritance patterns of AS. While the XL-AS exhibited gradually worsened hearing thresholds according to age, the thresholds did not differ over age in AD-AS; it is difficult to speculate on the trend in the change of the hearing threshold in AR-AS due to an insufficient number of cases (Figure 2B–D). This indicates the AD-AS subtype exhibits similar trends of progressive hearing loss, although the onset of significant hearing deterioration is seen slightly earlier than in XL-AS. Across all genetic subtypes, the hearing thresholds (measured in decibels hearing level, dB HL) were consistently higher in the older age groups.

Among the patients with XL-AS, the hearing thresholds were compared according to the sex, given that COL4A5 is located in the X chromosome; thus, the phenotype of males and females might be different. Although the number of female patients with XL-AS was limited, there was no difference in the hearing threshold in the PTA_4_ according to sex. This finding indicates that XL-AS does not exhibit different penetrance and phenotype depending on sex.

## 4. Discussion

The natural history of hearing loss in AS represents a vital aspect of the clinical course of the disease, alongside renal and ocular complications. Our study sheds light on the progression of SNHL across different genetic subtypes of AS, with particular emphasis on the XL-AS, AR-AS, and AD-AS forms. The findings provide essential insights into the timing, severity, and variability of hearing loss, which have important implications for clinical management and genetic counseling.

Earlier studies have demonstrated that the timing of hearing loss correlates with the specific genotype, with males with *COL4A5* mutations (XL-AS) often developing SNHL earlier than those with *COL4A3* or *COL4A4* mutations (ARAS) [14,30]. Females with XLAS and individuals with AD-AS typically experience later-onset and less severe hearing loss due to the partial expression of functional collagen proteins. The majority of patients in this cohort were diagnosed with XL-AS, which is consistent with the literature that reports the X-linked inheritance accounts for approximately 80% of AS cases [31,32]. Our results demonstrate that XL-AS is characterized by an early onset of hearing impairment, typically during the second decade of life. This finding is supported by studies that identify mutations in the *COL4A5* gene as the primary cause of early cochlear basement membrane dysfunction [19].

**Figure 3:**
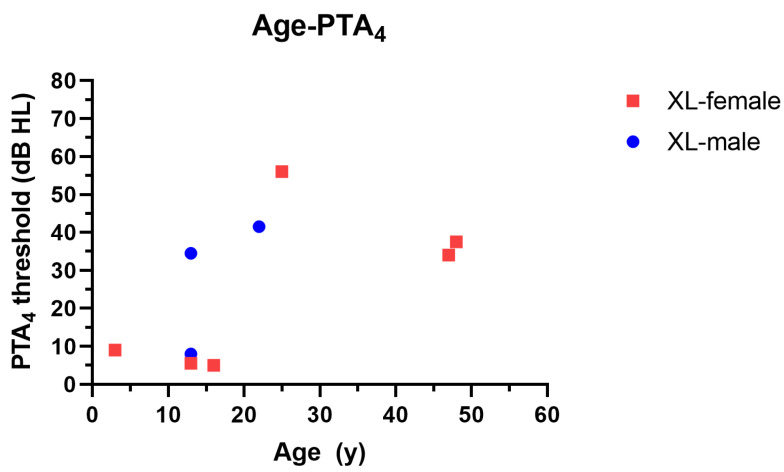
Difference in hearing threshold according to sex in XL-AS. Auditory thresholds are compared depending on sex among nine patients with XL-AS. There is no remarkable difference in hearing impairment between male and female individuals. XL, X-linked type.

Hearing loss in these patients is progressive, often starting with high-frequency thresholds and gradually affecting lower frequencies, a typical pattern in sensorineural deafness associated with cochlear pathology [30,33]. Previous research has described this progression, highlighting that male patients with XL-AS are more likely to experience earlier and more common hearing impairment than their female counterparts [20,30,34]. However, our study revealed no remarkable sex-related differences in terms of the severity of hearing loss, which may be explained by the relatively small number of male patients and the variability in the X-chromosome inactivation in the female carriers (Figure 3) [14]. Notably, the progression of hearing loss in patients with AS follows a nonlinear pattern, with a plateau in the auditory thresholds observed around 53 dB HL. This plateau suggests that hearing loss in these patients, while progressive, does not typically worsen beyond a moderate hearing loss level. Nevertheless, the progressive nature of the impairment underscores the importance of regular audiological follow-up even in cases of mild initial hearing loss.

The progressive nature of SNHL in AS, particularly in XL-AS, emphasizes the importance of early diagnosis and intervention. Early identification of hearing impairment through audiometric screening is crucial for initiating timely interventions that may delay or mitigate further auditory deterioration [30]. This is particularly relevant for pediatric populations, where undiagnosed hearing loss can significantly impact language development, educational outcomes, and overall quality of life [35].

For patients with AS, the choice of auditory rehabilitation strategies should be tailored to the individual’s genetic background and the severity of the hearing loss. Hearing aids are often the first line of intervention in patients with mild to moderate SNHL. When hearing loss progresses, cochlear implants may be considered, especially in cases where the hearing thresholds surpass 50–60 dB HL. However, auditory thresholds in patients with AS rarely surpass 50–60 dB HL; thus, it might be less common for these patients to need cochlear implantation. In our study, none of the patients had reached the threshold for cochlear implantation, but continued monitoring is essential to determine the optimal timing for such interventions. Furthermore, our findings highlight the necessity of personalized management plans that consider the inheritance pattern and specific genetic mutations. Genetic testing plays a pivotal role in diagnosing AS, guiding clinical decisions, and providing prognostic information.

While our study provides valuable insights into the natural history of hearing loss in AS, the relatively small sample size, particularly in the AR-AS and AD-AS groups, limits the generalizability of our findings. In particular, the auditory thresholds at different ages were not paired data, which may raise concerns about potential unintentional bias in the nonlinear correlation analyses. Therefore, larger multi-center studies are needed to confirm the trends of progressive hearing loss according to age observed in our cohort and provide a more comprehensive understanding of the auditory phenotypes in different genetic subtypes of AS.

In conclusion, our study confirms that hearing loss in AS is early-onset and progressive but does not surpass the moderate level of hearing loss. These findings underscore the importance of early diagnosis, regular audiometric monitoring, and personalized rehabilitation strategies.

## Figures and Tables

**Table 1:** Demographics of the enrolled patients (n = 15).

Characteristics	Value (%)
Age (yr)	38.7 ± 19.9
Sex	
Male	4 (26.7)
Female	11 (73.3)
Age of onset	
1st decade	2 (13.3)
2nd decade	8 (53.3)
3rd decade	1 (6.7)
4th decade	2 (13.3)
5th decade	2 (13.3)
Pure tone average (dB HL)	
Right	31.3 ± 19.6
Left	38.2 ± 20.2
Hearing loss severity	
Normal (0–25 dB HL)	4 (26.7)
Mild (25–39 dB HL)	2 (13.3)
Moderate (40–69 dB HL)	9 (60.0)
Pure tone audiometry pattern	
Flat-type	15 (100.0)

**Table 2:** Genetic variants of patients diagnosed with AS.

Individual	Age (Year)	Sex	Inheritance Pattern	Hematuria	Proteinuria	PTA_4_ (dB HL)	Gene	Nucleotide Change	Amino Acid Change	Zygosity	SIFT	Polyphen-2	Mutation Taster	PhyloP	GERP^++^	CADD Phred	DVD Classification	ACMG/AMP Reference or PMID
1	19	F	AD	+	+	44.5	COL4A3	c.1029+1G>A	Splicing donor variant	Het	−	−	−	−	−	34	VUS	Pathogenic (PVS1, PS2, PS4, PM2)
2	56	F	AD	−	−	52	COL4A3	c.3499G>A	p.G1167R	Het	D	PD	DC	C	C	32	Pathogenic	11134255, 14582039, 28542346
3	55	M	AD	−	−	52.5	COL4A3	c.1295C>T	p.P432L	Het	D	PD	DC	NC	C	22.7	Pathogenic	23967202
4	18	F	AD	+	+	39.5	COL4A4	c.4901G>C	p.C1634S	Het	D	PD	DC	C	C	27.8	Pathogenic	15086897
5	38	F	AR	−	+	44.0	COL4A3	c.4793T>G	p.L1598R	Homo	D	PD	DC	C	C	29.4	Pathogenic	25741868,24633401,28492532
6	44	F	AR	−	−	58.5	COL4A4	c.2869G>A	p.G957R	Comp. Het	D	PD	DC	C	C	24.9	Pathogenic	27281700
								c.2045A>G	p.D682G		T	PD	Poly	C	C	22.2	Pathogenic	19675380, 23967202, 25741868
7	48	F	XL	−	+	37.5	COL4A5	c.511G>A	p.G171S	Hemi	D	PD	DC	C	C	23.7	Pathogenic	26467025
8	25	F	XL	−	−	56.0	COL4A5	c.3985C>T	p.P1329S	Hemi	T	B	DC	C	C	21.0	VUS	Likely pathogenic (PS4, PM2, PM5)
9-1 9-2	13 47	M F	XL	+ +	+ +	34.5 34.0	COL4A5	c.3860_3861dupAA	p.G1288Kfs*18	Hemi	−	−	−	−	−	−	−	Pathogenic (PVS1, PS4, PM2, PP1)
10-1 10-2	13 16	M F	XL	– –	+ +	8 5	COL4A5	c.511G>A;	p.G171S	Hemi	D	PD	DC	C	C	23.7	Pathogenic	26467025
11	13	F	XL	–	+	5.5	COL4A5	c.3958C>T	p.P1320S	Hemi	D	PD	DC	C	C	23.7	Pathogenic	14993485,26467025,28492532
12	3	F	XL	+	+	9.0	COL4A5	c.584G>T	p.G195V	Hemi	D	PD	DC	C	C	26.1	Pathogenic	Likely pathogenic (PS2, PM2, PM5)
13	22	M	XL	−	+	41.5	COL4A5	c.1589G>T	p.G530V	Hemi	D	PD	DC	C	C	32	Pathogenic	21143337

AD, autosomal dominant; AR, autosomal recessive, XL, X-linked; Comp., compound; D, damaging; DC, disease causing; C, conserved; PTA_4_, pure-tone average threshold of both ears (1, 2, 3, and 4 KHz).

## Data Availability

Data are contained within the article.

## References

[1] Kashtan C.E. (2021). Alport syndrome: Achieving early diagnosis and treatment. Am. J. Kidney Dis..

[2] Warady B.A., Agarwal R., Bangalore S., Chapman A., Levin A., Stenvinkel P., Toto R.D., Chertow G.M. (2020). Alport syndrome classification and management. Kidney Med..

[3] McCarthy P.A., Maino D.M. (2000). Alport syndrome: A review. Clin. Eye Vis. Care.

[4] Gubler M., Levy M., Broyer M., Naizot C., Gonzales G., Perrin D., Habib R. (1981). Alport’s syndrome: A report of 58 cases and a review of the literature. Am. J. Med..

[5] Barozzi S., Soi D., Intieri E., Giani M., Aldè M., Tonon E., Signorini L., Renieri A., Fallerini C., Perin P. (2020). Vestibular and audiological findings in the Alport syndrome. Am. J. Med. Genet. Part A.

[6] Pedrosa A.L., Bitencourt L., Paranhos R.M., Leitáo C.A., Ferreira G.C., Simões e Silva A.C. (2021). Alport syndrome: A comprehensive review on genetics, pathophysiology, histology, clinical and therapeutic perspectives. Curr. Med. Chem..

[7] Kruegel J., Rubel D., Gross O. (2013). Alport syndrome—Insights from basic and clinical research. Nat. Rev. Nephrol..

[8] Longo I., Porcedda P., Mari F., Giachino D., Meloni I., Deplano C., Brusco A., Bosio M., Massella L., Lavoratti G. (2002). COL4A3/COL4A4 mutations: From familial hematuria to autosomal-dominant or recessive Alport syndrome. Kidney Int..

[9] Kashtan C.E., Gross O. (2021). Clinical practice recommendations for the diagnosis and management of Alport syndrome in children, adolescents, and young adults–an update for 2020. Pediatr. Nephrol..

[10] Chavez E., Rodriguez J., Drexler Y., Fornoni A. (2022). Novel therapies for Alport syndrome. Front. Med..

[11] Gross O., Kashtan C.E. (2009). Treatment of Alport syndrome: Beyond animal models. Kidney Int..

[12] Harvey S.J., Zheng K., Sado Y., Naito I., Ninomiya Y., Jacobs R.M., Hudson B.G., Thorner P.S. (1998). Role of distinct type IV collagen networks in glomerular development and function. Kidney Int..

[13] Cosgrove D., Liu S. (2017). Collagen IV diseases: A focus on the glomerular basement membrane in Alport syndrome. Matrix Biol..

[14] Nozu K., Nakanishi K., Abe Y., Udagawa T., Okada S., Okamoto T., Kaito H., Kanemoto K., Kobayashi A., Tanaka E. (2019). A review of clinical characteristics and genetic backgrounds in Alport syndrome. Clin. Exp. Nephrol..

[15] Satoh H., Kawasaki K., Kihara I., Nakano Y. (1998). Importance of type IV collagen, laminin, and heparan sulfate proteoglycan in the regulation of labyrinthine fluid in the rat cochlear duct. Eur. Arch. Oto-Rhino-Laryngol..

[16] Gratton M.A., Rao V.H., Meehan D.T., Askew C., Cosgrove D. (2005). Matrix metalloproteinase dysregulation in the stria vascularis of mice with Alport syndrome: Implications for capillary basement membrane pathology. Am. J. Pathol..

[17] Yang W., Zhao X., Chai R., Fan J. (2023). Progress on mechanisms of age-related hearing loss. Front. Neurosci..

[18] Tryggvason K., Zhou J., Hostikka S.L., Shows T.B. (1993). Molecular genetics of Alport syndrome. Kidney Int..

[19] Hudson B.G., Tryggvason K., Sundaramoorthy M., Neilson E.G. (2003). Alport’s syndrome, Goodpasture’s syndrome, and type IV collagen. N. Engl. J. Med..

[20] Jais J.P., Knebelmann B., Giatras I., De Marchi M., Rizzoni G., Renieri A., Weber M., Gross O., Netzer K.-O., Flinter F. (2003). X-linked Alport syndrome: Natural history and genotype-phenotype correlations in girls and women belonging to 195 families: A “European Community Alport Syndrome Concerted Action” study. J. Am. Soc. Nephrol..

[21] Lujinschi Ș.N., Sorohan B.M., Obrișcă B., Vrabie A., Lupușoru G., Achim C., Andronesi A.G., Covic A., Ismail G. (2024). Genotype–Phenotype Correlations in Alport Syndrome—A Single-Center Experience. Genes.

[22] Joo S.Y., Jang S.H., Kim J.A., Kim S.J., Kim B., Kim H.-Y., Choi J.Y., Gee H.Y., Jung J. (2023). Prevalence and clinical characteristics of mitochondrial DNA mutations in Korean patients with sensorineural hearing loss. J. Korean Med. Sci..

[23] Rim J.H., Noh B., Koh Y.I., Joo S.Y., Oh K.S., Kim K., Kim J.A., Kim D.H., Kim H.Y., Yoo J.E. (2022). Differential genetic diagnoses of adult post-lingual hearing loss according to the audiogram pattern and novel candidate gene evaluation. Hum. Genet..

[24] Song M.H., Jung J., Rim J.H., Choi H.J., Lee H.J., Noh B., Lee J.S., Gee H.Y., Choi J.Y. (2020). Genetic inheritance of late-onset, down-sloping hearing loss and its implications for auditory rehabilitation. Ear Hear..

[25] Oh K.S., Walls D., Joo S.Y., Kim J.A., Yoo J.E., Koh Y.I., Kim D.H., Rim J.H., Choi H.J., Kim H.-Y. (2022). COCH-related autosomal dominant nonsyndromic hearing loss: A phenotype–genotype study. Hum. Genet..

[26] Joo S.Y., Na G., Kim J.A., Yoo J.E., Kim D.H., Kim S.J., Jang S.H., Yu S., Kim H.-Y., Choi J.Y. (2022). Clinical heterogeneity associated with MYO7A variants relies on affected domains. Biomedicines.

[27] Lee H.J., Lee J.M., Na G., Moon Y.M., Lee C., Jung J. (2020). Which patients with a unilateral hearing aid for symmetric sensorineural hearing loss have auditory deprivation?. Clin. Exp. Otorhinolaryngol..

[28] Jung J., Jang S.H., Won D., Gee H.Y., Choi J.Y., Jung J. (2024). Clinical Characteristics and Audiological Profiles of Patients with Pathogenic Variants of WFS1. J. Clin. Med..

[29] Oza A.M., DiStefano M.T., Hemphill S.E., Cushman B.J., Grant A.R., Siegert R.K., Shen J., Chapin A., Boczek N.J., Schimmenti L.A. (2018). Expert specification of the ACMG/AMP variant interpretation guidelines for genetic hearing loss. Hum. Mutat..

[30] Jais J.P., Knebelmann B., Giatras I., De Marchi M., Rizzoni G., Renieri A., Weber M., Gross O., Netzer K.-O., Flinter F. (2000). X-linked Alport syndrome: Natural history in 195 families and genotype-phenotype correlations in males. J. Am. Soc. Nephrol..

[31] Kashtan C.E., Michael A.F. (1996). Alport syndrome. Kidney Int..

[32] Chavez E., Goncalves S., Rheault M.N., Fornoni A. (2024). Alport Syndrome. Adv. Kidney Dis. Health.

[33] Aldè M., Cantarella G., Zanetti D., Pignataro L., La Mantia I., Maiolino L., Ferlito S., Di Mauro P., Cocuzza S., Lechien J. (2023). Autosomal Dominant Non-Syndromic Hearing Loss (DFNA): A Comprehensive Narrative Review. Biomedicines.

[34] Savige J., Colville D., Rheault M., Gear S., Lennon R., Lagas S., Finlay M., Flinter F. (2016). Alport syndrome in women and girls. Clin. J. Am. Soc. Nephrol..

[35] Shojaei E., Jafari Z., Gholami M. (2016). Effect of early intervention on language development in hearing-impaired children. Iran. J. Otorhinolaryngol..

